# Virtual Interviews Improve Equity and Wellbeing: Results of a Survey of Applicants to Obstetrics and Gynecology Subspecialty Fellowships

**DOI:** 10.1186/s12909-022-03679-y

**Published:** 2022-08-15

**Authors:** Ann Do Tran, Christine A. Heisler, Sylvia Botros-Brey, Hanzhang Wang, Bertille Gaigbe-Togbe, Ava Leegant, Anne Hardart

**Affiliations:** 1 Mount Sinai Hospital Icahn School of Medicine at Mount Sinai, 1176 Fifth Ave, KP9, New York, NY 10029 USA; 2 Meriter Hospital University of Wisconsin School of Medicine and Public Health, 202 S. Park, Madison, WI 53715 USA; 3 The University of Texas at San Antonio Joe R. & Teresa Lozano Long School of Medicine , 7703 Floyd Curl Drive, San Antonio, TX 78229 USA; 4 Montefiore Medical Park Albert Einstein College of Medicine, 1695 Eastchester, Road Bronx, NY 10461 USA

**Keywords:** Virtual interviews, Equity, Wellbeing, Subspecialty training, Obstetrics and Gynecology

## Abstract

**Background:**

Nationwide restrictions and recommendations from the Association of American Medical Colleges mandated program directors to conduct all graduate medical education interviews virtually in the Spring of 2020 in response to the COVID-19 pandemic. This study was conducted to assess the impact of virtual interviews on a candidates’ ability to effectively create a rank list.

**Objective:**

The primary objective of this study was to evaluate Obstetrics and Gynecology (ObGyn) subspecialty fellowship applicants’ perspectives regarding the effectiveness of virtual interviews for creating a rank list. Secondary outcomes included perceived advantages and disadvantages of the process and costs of the process.

**Methods:**

This was a cross-sectional IRB-exempt study, using an electronic survey administered to a convenience sample of applicants to ObGyn subspecialty fellowship programs. The survey was administered via RedCap between the rank list submission deadline and the Match. Descriptive statistics were used.

**Results:**

Response rate was 158/330 (48%). Overall, 129/158 (82%) percent of respondents felt confident in making their rank list based on the virtual interviews, and 146/158 (92%) were “very satisfied” or “somewhat satisfied” with the process. Of those who expressed an interview style preference, 65/149(44%) of respondents preferred virtual interviews; 49/149(33%) had no preference or were not sure. Nearly all 146/148(99%) applicants cited cost-savings as a distinct advantage of virtual interviews.

**Conclusion:**

Applicants to ObGyn subspecialty fellowships felt comfortable to create a rank list based on the virtual interview. This study indicates that the virtual format is effective, less stressful and less costly for ObGyn subspecialty interviews and should be considered beyond the pandemic to remove barriers and burdens for applicants.

## Background

Interviews are an important component of the fellowship application process and remain a key factor that helps programs in selecting future fellows. [1] ObGyn interviews have historically been in-person, which is a costly and time-intensive tradition. [2–4] Applicants use interviews to showcase their strengths, learn about specific programs, meet faculty, fellows and other applicants, and visit the facilities and the locations of the programs. Consideration of alternatives to in-person interviews have been proposed prior to the COVID-19 pandemic, including hybrid virtual screening interviews.[5–7] However, small survey studies of applicants in single specialties have shown conflicting results regarding applicants’ perspectives on virtual interviews. [8–10]

During the early Coronavirus Disease 19 (COVID-19) pandemic entire subspecialties conducted interviews virtually out of necessity.[2, 11] Within ObGyn, Female Pelvic Medicine and Reconstructive Surgery (FPMRS) was the first subspecialty to adapt to an entirely virtual platform for interviews due to the early match cycle. A survey of FPMRS program directors found virtual interviews effective in evaluating applicants, and most were satisfied with the process.[12 A subsequent study of FPMRS applicants noted a positive experience with the virtual format, and 83.3% were confident in ranking programs based on this experience.[13] Virtual interview strategies and lessons learned by FPMRS programs were implemented by other ObGyn subspecialty fellowship program directors participating in the later Match cycle. A study of Maternal Fetal Medicine applicants found that the virtual interview format was viewed favorably and suggested that insights gained should inform future application cycles.[14]^.^

All programs, however, adopted the virtual interview process independently. Given the importance of the interview process, this study was conducted to determine whether applicants to ObGyn subspecialties perceived the virtual interview structure provided enough information to create the rank list. Secondary objectives included preferences for interview type (virtual vs. in-person), perceived advantages and disadvantages of virtual interviews, and costs of the interview process.

## Methods

This study is a cross-sectional internet-based survey of applicants to ObGyn fellowship programs who interviewed during 2020 for the 2021 National Resident Matching Program. Specific survey questions were developed based on the general themes obtained from an anonymous questionnaire to FPMRS applicants. The survey consisted of multiple-choice questions with Likert scale response format and yes/no questions, including a “prefer not to answer” or “not sure” option for most questions (link to supplemental digital content with Survey). Applicants were queried regarding their satisfaction with the virtual interviews and preparedness to rank fellowship programs. Additional questions pertained to their preferences for specific virtual platforms, experience with additional interview components, perceived advantages and disadvantages of virtual interviews, financial expenses, impact on residency duties, and stressfulness of the process. Applicants were prompted to compare the virtual interview experience to in-person residency interviews. Demographic data included age, gender, race/ethnicity, type of residency program and geographic regions. One free-response prompt was included at the end of the survey to obtain additional constructive feedback regarding the virtual interview process.

The survey was developed using Research Electronic Data Capture platform (REDCap), a web-based software platform designed to support data capture for research studies.[15] The electronic form was reviewed for readability and flow by the authors, internally validated with FPMRS fellows, reviewed by collaborating program directors and revised. The survey was initially piloted with FPMRS subspecialty applicants and reviewed by members of the Council of Fellowship Training in Obstetrics and Gynecology (COFTOG).[13] In preparation for distribution to additional subspecialty applicants, minor revisions were made to the initial survey, including subspecialty, region of residence, and dollar amount spent. The Checklist for Reporting Results of Internet ESurveys (CHERRIES) was utilized for this survey.[16]

Study data were collected and sent through links to Mount Sinai REDCap. The survey link was available only between the dates of the rank list submission deadline and Match day, in order to avoid the perception that the survey might affect the match results. Participation was voluntary and anonymous, and consent was obtained as part of the survey process. This study was granted exemption status by the Mount Sinai Institutional Review Board.

The survey was initially sent to FPMRS applicants and then to a convenience sample of applicants in Maternal Fetal Medicine (MFM), Gynecologic Oncology, Complex Family Planning and Minimally Invasive Gynecologic Surgery. After obtaining approval from program directors at the investigator’s institutions, applicants’ email addresses were obtained in each subspecialty and an email with a link to the survey was sent. Given privacy constraints and specific dates of survey distribution, candidates from the Reproductive Endocrinology and Infertility programs were unable to be included.

Two sample t-test and Wilcoxon rank-sum test were used for comparing continuous variables, while categorical variables were compared using chi-square and one-way ANOVA test. Statistical significance was defined as *p* < 0.05. Data were analyzed using Stata statistical software (Release 15. College Station, TX).

## Results

A total of 330 surveys were delivered via email to applicants. Of these, 158/330 (47.9%) responded, and 149 responded to demographic questions. Because respondents were permitted to omit questions, the denominators varied among questions. The majority of respondents were women 122/149 (81.9%), White 85/158(53.8%) and 30–34 years of age 86/149(57.7%) (Table 1). Geographic location was evenly distributed across the Northeast, Midwest and South, with less representation from the West. Most candidates who responded were applying to Maternal Fetal Medicine (MFM) (47/146, 32.3%) and FPMRS (40/150, 26.6%).

**Table 1:** Demographic data based on Fellowship Sub-Specialty, N (%)

	**Complex family planning**	**Female pelvic medicine and reconstructive surgery**	**Gynecologic oncology**	**Maternal fetal medicine**	**Minimally invasive gynecologic surgery**	**Total *n* = 149**	
**Gender**							
Female	16 (94.1)	31 (77.5)	15 (83.3)	37 (78.7)	23 (85.2)	122(81.9%)	
Male	1 (5.9)	8 (20.0)	2 (11.1)	10 (21.3)	4 (14.8)	25(16.8%)	
Non-binary	0	0	0	0	0	0	
Prefer Not To Answer	0 (0)	1 (2.5)	1 (5.6)	0 (0)	0	2(1.3%)	
Race and ethnicity*							
Asian	3 (23.1)	8 (21.1)	4 (22.2)	10(20.9)	9 (36.0)	33/149 (22.1)	
Black	1 (7.7)	1 (2.6)	0 (0)	3 (7.0)	3 (12.0)	8 (5.4)	
White	9 (69.2)	24 (63.2)	11 (61.1)	29 (67.4)	12 (48.0)	87 (58.3)	
Native American	0	0	0	1	0	1 (0.6)	
other	0	1	0	1	0	2(1.3)	
Prefer Not To Answer	0 (0)	4 (10.5)	1 (5.6)	0 (0)	0 (0)	5(3.3)	
Hispanic	4 (22.2)	2 (4.8)	0 (0)	8 (15.7)	4 (13.8)	18	
*Respondents could choose more than one answer **Age (Years)**							
< 30	5 (29.4)	15 (37.5)	6 (33.3)	19 (40.4)	7 (25.9)	52/149(34.9)	
30–34	11 (64.7)	23 (57.5)	11 (61.1)	24 (51.1)	17 (63)	86/149(57.7)	
35–39	1 (5.9)	1 (2.5)	0 (0)	4 (8.5)	3 (11.1)	9/149 (6.0)	
> 40	0	0	0	0	0	0	
Prefer Not To Answer	0 (0)	1 (3)	1 (6)	0 (0)	0 (0)	2/149 (1.3)	
**Geographic Region *n* = 106**							
Midwest	6/17 (35.3)	N/A*	6/17 (35.3)	10/46(21.7)	6/26 (23.1)	28 (26.1)	
Northeast	4 (23.5)	N/A	6 (35.3)	20 (43.5)	8 (30.8)	38 (35.5)	
South	3 (17.6)	N/A	4 (23.5)	13 (28.3)	7 (26.9)	27 (25.2)	
West	4 (23.5)	N/A	0 (0)	3 (6.5)	3 (11.5)	10 (9.3)	
Prefer Not To Answer	0 (0)	N/A	1 (5.9)	0 (0)	2 (7.7)	3 (2.8)	

^*^Question was not asked in surveys for FPMRS candidates

Overall, 129/158 (82%) expressed confidence in making their rank list based on the virtual interviews, and 146/158 (92%) of respondents were “very satisfied” or “somewhat satisfied” with the process. Respondents felt they obtained adequate information about the fellowship programs 149/152(98%), program faculty 136/152(89%), and fellows 128/151 (85%) from virtual interviews. One-third 49/152 felt they were able to obtain an adequate perception of the locations as possible places to live and train. A large majority 136/150 (90.7%) found virtual interviews to be less stressful or about the same as in-person interviews.

Virtual interviews were preferred by 65/149 (44%) of respondents, although 49/149 (33%) had no preference or were unsure. Preference for interview type did not differ by gender or age, or race. Respondents residing in the Midwest were more likely to prefer virtual interviews 18/30(60%) compared to those from other regions (*p* = 0.03). Subspecialty was not associated with interview preference (*p* = 0.28). Nearly all respondents preferred Zoom as a virtual interview platform 138/151 (91%).

The associated advantages to virtual interviews included convenience, cost and time savings; unsurprisingly, disadvantages pertained to inability to tour the institution or community and lack of direct interactions (Table 2). In fact, nearly all candidates 146/148 (99%) reported lower cost to be the most significant advantage of the virtual interview platform (Table 2). To enumerate, half of the respondents (73/150) estimated in-person fellowship interview expenses to exceed $6,000 (Table 3). Over 20% (31/149) of applicants acknowledged they would have incurred additional debt through loans or credit cards in order to finance anticipated in-person interviews. However, the median (range) expenses in the virtual interview platform was merely $200 ($0-$3,500).

**Table 2:** Advantages and disadvantages for virtual interview experience

Advantages (*N* = 148)	*n* (%)	Disadvantages (*N* = 149)	*n* (%)
Reduced Cost	146 (98.6)	Inability to Visit/Tour the Institutions	118 (79.2)
Convenience	134 (90.5)	Inability to Interact with Co-Applicants	117 (78.5)
Time Saving	135 (91.2)	Inability to Interact Face-to-Face	108 (72.4)
Less Time Away from Clinical Duties	135 (91.2)	Inability to Visit Specific Geographic Locations	102 (68.5)
Flexibility of Scheduling Interviews	117 (79.1)	Inadequate Opportunity to Present Myself	56 (37.6)
Ability to Interview at More Programs	108 (73.0)	Technical Difficulties with Meeting Platforms	46 (30.9)
Other	1 (0.7)	Other	4 (2.7)
Prefer Not to Answer	0 (0)	Prefer Not to Answer	0 (0)
No Advantages	1 (0.7)	No Disadvantages	2 (1.3)

**Table 3:** Applicant-based recommendations for optimizing virtual interviews

Maximize the experience and minimize stress	· Provide ample opportunity to meet with current trainees and staff · Reassure faculty and applicants that interviews can be conducted by phone, in the event of loss of connection · Provide a separate “room” for socializing and asking questions · Solicit feedback after the interviews
Improve applicants’ ability to present themselves	· Provide applicants with AAMC applicant preparation guide for virtual interviews
Improve face-to-face interactions	· Allow breaks between interviews · Minimize distractions by silencing phones, etc
Improve ability to interact with other applicants	· Plan pre-interview social activities that end at a fixed time · Allow time for applicants to congregate without faculty · Encourage social media connections after interviews
Improve applicants’ ability to get a sense of location and facilities	· Showcase facilities with virtual tours · Supply information ahead of time about: program, institution, location or city, cost of living, types of recreation and entertainment · Encourage discussion with current trainees specifically about locale, community and lifestyle
Minimize bias, improve equity	· If offering virtual interviews, do it uniformly to maintain equity · Reinforce implicit bias training for all interviewers · Encourage virtual backgrounds to minimize bias · Pay attention to time zone differences for applicants · Consider standardized questions and scoring rubric
Minimize technical difficulties	· Appoint a tech-savvy moderator · Test platform with interviewers prior to interview day · Offer to test technology with applicants prior to interviews · Consider a platform like Zoom, which was favored by applicants · Provide contact numbers to call in case of technical difficulties · Ensure interviewers are aware of potential technological difficulties/inequities and do not penalize applicants for them

## Discussion

This is a national multi-specialty survey of ObGyn fellowship applicants conducted to determine whether the virtual interview process was effective for the purposes of creating a rank list. Our data indicate that a majority of interviewees 129/158(81.6%) for ObGyn subspecialty fellowships found that the virtual interview process provided enough information and gave an adequate impression of the programs to inform the applicants’ ability to create rank lists. Almost all respondents 146/148 (98.6%) cited cost savings as an advantage, and a large majority found virtual interviews to be less stressful or about the same 136/150 (90.7%) as in-person interviews.

While virtual interviewing has previously been used in smaller settings, 2020 was the first year it was adopted on such a large scale due to COVID restrictions, raising the question of its effectiveness for ranking in the subspecialty match. The applicant perspective for FPMRS applicants, who interviewed during the early phase of the pandemic, showed similar results, with overall ability to create a rank list, satisfaction with the process, and advantages of decreased cost and stressfulness. [13] Ding et al. also surveyed applicants to multiple subspecialities and found strengths of the virtual platform included cost savings and ease of scheduling interviews but noted uncertainty in ability to create a rank list. [17] Our larger and broader study corroborates some of their findings, including the clear financial benefit, but found virtual interviews to be adequate in creating a rank list.

Across all subspecialties, nearly all applicants 146/158(92.4%) were satisfied or very satisfied with the process, though responses were divided regarding preferences for interview format. Given the unfamiliarity with and variability in virtual interview format, however, it is understandable that applicants were not as willing to commit to a preference. Applicants from the Midwest were more likely than those from other regions to prefer the virtual platform, perhaps due to significant travel distances and, thus, higher expenses for in-person interviews.

Importantly, 69% of respondents noted an overall decrease in stressfulness of virtual interviews compared with in-person interviews. The general perception that virtual interviews are less stressful may have implications for resident wellbeing. Increased debt among residents has also been found to correlate with lower quality of life and higher rates of burnout. [18] Although socioeconomic status of residents is somewhat difficult to determine, many residents are burdened with debt, and a recent AAMC report showed that the average debt of residents is $200,000. [19] Our data suggest that 20% of applicants to fellowship considered taking on additional debt to apply to fellowship with traditional in-person format.

As institutions strive to reduce stress and prevent burnout among trainees, consideration of converting to virtual interviews could help mitigate stress and improve wellbeing in residents considering fellowship. The anticipated expenses of in-person interviews were similar to those reported by other specialties. [2, 4, 17, 20] Nearly all applicants cite the dramatic reduction in actual expenses for virtual interviews as a significant advantage. Tseng et al. found that fellowship candidates during COVID 19 saved close to $6,000 in interview travel expenses. [21] In 2014, Iqbal et al. reported that the amount spent on the interview process was the only predictor of matching versus not matching to ObGyn subspecialties, with those spending more having a higher rate of matching.^19^ Lower costs may reduce the debt burden for applicants and also attract residents who might not otherwise consider applying to fellowships; the shift to virtual interviews could thus theoretically improve diversity among fellowship applicants.

There are many strengths of our study, including the broad range of ObGyn subspecialties and the wide range of applicants to several programs, encompassing a nationwide sample of applicants to ObGyn subspecialties. Most previous surveys of applicants, in contrast, have surveyed applicants to single institutions or specialties and consisted of smaller sample sizes or lower response rate. [17, 22] Importantly, our survey was developed by a team of investigators, reviewed by members of COFTOG, administered for accuracy to an internal group of fellows, and piloted on the subset of subspecialty applicants in FPMRS, who were in an earlier match cycle. The anonymous and voluntary survey, which underwent rigorous development by the investigators, followed the CHERRIES criteria. The timing of the survey, administered after match lists had been submitted and before the Match results were released, was planned to eliminate bias and concerns that responses could affect match results and vice versa.

This study has a few limitations. While a response rate of 48% may be considered low, it is actually reasonable for an online survey of physicians, especially given its short time frame for administration and the survey and email fatigue that may have been more pervasive during the pandemic. [23] A convenience sample was utilized for this study, with surveys sent only to applicants in subspecialties whose program directors were willing to participate and provide applicant contacts, thus perhaps introducing a sampling bias. The survey inquired about time away from work but did not specifically ask about the effect of virtual versus in-person interviews on family responsibilities, which may have posed an additional barrier for applicants. Respondents were allowed to omit questions, perhaps introducing response bias. Finally, lack of data on applicants to Reproductive Endocrinology and Infertility and Pediatric and Adolescent Gynecology limits generalizability to those fields.

As the graduate medical education (GME) community prepares for future fellowship interview seasons that may require a virtual platform, the process will be more familiar. Moreover, this generation of trainees, who carry significant financial debt and have professional and personal time constraints, are already accustomed to virtual meetings. Surveying all graduating residents would give a clearer picture of how the cost of interviewing affects the decision to apply or not. Suggestions for addressing the perceived disadvantages of virtual interviews, based on responses in this study, are listed in Table 3.

## Conclusions

Our results demonstrate that virtual interviews enabled applicants to effectively create their rank lists, and nearly all respondents were satisfied with the interview process, citing cost savings and decreased stressfulness as advantages. A study surveying this historic cohort of trainees who interviewed for residency in the in-person setting, and for fellowship in the virtual setting, is currently underway. From the perspective of the applicants, the benefits outweigh the disadvantages of virtual interviews and level the playing field. Fellowship program directors have an opportunity and perhaps obligation to consider this paradigm shift as a means to alleviate the emotional and financial burdens on applicants as well as to encourage all potential candidates, including those with fewer resources and those underrepresented, to pursue further training .

## Authors’ information

Ann Tran, MD.

Assistant Program Director, Female Pelvic Medicine and Reconstructive Surgery Fellowship.

Assistant Professor, Department of Obstetrics and Gynecology.

Icahn School of Medicine at Mount Sinai.

Christine Heisler, MD, MS.

Division Chief, Female Pelvic Medicine and Reconstructive Surgery.

Fellowship Program Director, Female Pelvic Medicine and Reconstructive Surgery.

Assistant Professor, Department of Obstetrics and Gynecology.

University of Wisconsin-Madison School of Medicine and Public Health.

Sylvia Botros-Brey, MD, MSCI.

Fellowship Program Director, Female Pelvic Medicine and Reconstructive Surgery.

Associate Professor, Department of Urology and Obstetrics and Gynecology.

University of Texas Health San Antonio Long School of Medicine.

Ava Leegant, MD.

Division Director, Female Pelvic Medicine and Reconstructive Surgery.

Fellowship Program Director, Female Pelvic Medicine and Reconstructive Surgery.

Associate Professor, Department of Urology and Obstetrics and Gynecology.

Albert Einstein College of Medicine.

Anne Hardart, MD.

Director of Gynecology, Mount Sinai West.

Fellowship Program Director, Female Pelvic Medicine and Reconstructive Surgery.

Associate Professor, Department of Obstetrics and Gynecology.

Icahn School of Medicine at Mount Sinai.

## Data Availability

The datasets used and/or analysed during the current study are available from the corresponding author on reasonable request.
